# The ultrastructure of book lung development in the bark scorpion *Centruroides gracilis *(Scorpiones: Buthidae)

**DOI:** 10.1186/1742-9994-8-18

**Published:** 2011-07-27

**Authors:** Roger D Farley

**Affiliations:** 1 Department of Biology, University of California, Riverside, California, 92521, USA

## Abstract

**Background:**

Near the end of the nineteenth century the hypothesis was presented for the homology of book lungs in arachnids and book gills in the horseshoe crab. Early studies with the light microscope showed that book gill lamellae are formed by outgrowth and possibly some invagination (infolding) of hypodermis (epithelium) from the posterior surface of opisthosomal limb buds. Scorpion book lungs are formed near the bilateral sites of earlier limb buds. Hypodermal invaginations in the ventral opisthosoma result in spiracles and sac-like cavities (atria). In early histological sections of embryo book lungs, widening of the atrial entrance of some lamellae (air channels, air sacs, saccules) was interpreted as an indication of invagination as hypothesized for book gill lamellae. The hypodermal infolding was thought to produce the many rows of lamellar precursor cells anterior to the atrium. The ultrastructure of scorpion book lung development is compared herein with earlier investigations of book gill formation.

**Results:**

In scorpion embryos, there is ingression (inward migration) of atrial hypodermal cells rather than invagination or infolding of the atrial hypodermal layer. The ingressing cells proliferate and align in rows anterior to the atrium. Their apical-basal polarity results in primordial air channels among double rows of cells. The cuticular walls of the air channels are produced by secretion from the apical surfaces of the aligned cells. Since the precursor cells are in rows, their secreted product is also in rows (i.e., primordial air channels, saccules). For each double row of cells, their opposed basal surfaces are gradually separated by a hemolymph channel of increasing width.

**Conclusions:**

The results from this and earlier studies show there are differences and similarities in the formation of book lung and book gill lamellae. The homology hypothesis for these respiratory organs is thus supported or not supported depending on which developmental features are emphasized. For both organs, when the epithelial cells are in position, their apical-basal polarity results in alternate page-like channels of hemolymph and air or water with outward directed hemolymph saccules for book gills and inward directed air saccules for book lungs.

## Background

At the end of the ninteenth century and in the early twentieth century numerous papers were published comparing the development of book gills in the horseshoe crab with the development of book lungs in arachnids, especially spiders. As reviewed by Farley [1], this work was done with the hypothesis that these respiratory structures are homologous, e.g., the internal book lungs in the opisthosoma were derived by insinking of the anlage that had previously resulted in external book gills of an aquatic ancestor. There have been diagrams and much discussion about how an ancient ancestor with lamellate gills like extant horseshoe crabs could have given rise to arachnid book lungs [2-12]. Recent investigations of horseshoe crab, scorpion and spider embryos report similar patterns of gene expression at the bilateral opisthosomal sites where book gills or book lungs eventually form [13-17].

In early studies with the light microscope and histological sections, the air sacs (air channels, lamellae, saccules) of developing spider and scorpion book lungs were suggested to be infoldings of the hypodermis from the spiracular invagination (primordial atrium) posterior to opisthosomal limb buds. This process was thought to be similar to the small amount of invagination that may occur along with outgrowth folds for book gill development at the posterior surface of branchial appendages in horseshoe crabs [2-10,18-22]. Slight widening of the air sac entrance at the atrial wall was interpreted as indications of hypodermal infolding. The presumed infoldings were thought to result in the parallel rows of lamellar precursor cells anterior to the atrium. In the spider species they examined, Montgomery [23] and Janeck [24] reported that the initial widenings of the air sac entrance are transitory, and the air sacs are formed from aligned cells in a cluster derived from the hypodermis.

More recently for the spider *Cupiennius salei *[25], the segment polarity gene *engrailed-1 *is reported to be expressed as five stripes at the site where lamellae originate posterior to the second opisthosomal limb buds. The primordial site is invaginated and covered ventrally by the posterior folding and flattening of the preceding limb bud, as reported in earlier histological studies [8,23]. Also in *C. salei*, the developmental gene *pdm/nubbin *is expressed in a striped pattern possibly related to lamellar formation [15].

In his diagram of histological sections of scorpion embryos, Brauer [19] showed some small folds in the atrial wall. This was considered as evidence of hypodermal invagination like that proposed for book gills [17,22,26] although the presumptive folds were not actually shown to be related to the formation of book lung lamellae.

As pointed out earlier [1], lamellate respiratory organs are important for our understanding of evolutionary history and taxonomic relationships, but modern procedures are needed for a more detailed comparison of cell activity during book gill and book lung development. The main objective herein is to use transmission electron microscopy (TEM) to examine cell ultrastructure during formation of scorpion book lungs. The results can then be used where relevant and helpful for evolutionary hypotheses and further comparative studies.

The scanning electron microscope (SEM) was used in recent developmental investigations of the respiratory organs in the scorpion [27] and horseshoe crab [1]; the present investigation is a continuation of that effort. The SEM study of book lung development in scorpions [27] provides an overview of the process, but the SEM is limited in the resolution of cell detail. Also, tissue preparation requires dissection and/or fracturing to expose components for viewing. This has potential for cell damage and/or loss, with emphasis on the surface features of the tissue or organ. In the present study, whole book lungs were removed, and sections were cut at successive stages of development in embryos and first and second instars.

Book lung formation in scorpions is a slow and gradual process [27]. It begins in the embryo with the appearance of a spiracle and a sac-like invagination (primordial atrium) just inside the spiracle. Lamellar development continues through birth and the first molt that occurs 1-2 weeks after the newborn first instars (pronymphs) climb up on the mother's back. The book lung gradually becomes a functional respiratory organ with about 50 lamellae in the active and foraging second instar. The book lung cuticle is replaced in subsequent molts [28], and lamellae are increased in size and number so there are more than 150 in the adult. The earlier [27] and present investigations show a complex developmental process of cell proliferation, migration, alignment and secretion of cuticular materials. The result is a stable and highly ordered series of page-like air and hemolymph channels.

Information about scorpion anatomy and morphology and the general organization of scorpion book lungs and horseshoe crab book gills is provided in earlier publications [1,29-31].

## Methods

Gravid females of *Centruroides gracilis *[32] were purchased from a supplier (Strictly Reptiles, Hollywood, FL). Information about taxonomy and life history is provided by Francke and Jones [33], Sissom and Lourenço [34], Ades [35] and Fet et al. [36]. For taxonomy of spider species mentioned herein, the new Platnick catalog [37] is available on-line.

Maintenance of animals and the dissection and preparation of tissues for SEM were done as described by Farley [27]. For TEM, microscissors and forceps were used to remove the spiracle, booklungs and attached soft cuticle from embryos, while only the spiracle and booklungs were removed from the first and second instars. Tissues were fixed 6-12 hours (23-25°C) in 2.5% glutaraldehyde in 0.1 M cacodylate buffer. The tissues were washed in 0.1 M buffer and postfixed (1-2 days) in 1% OsO_4 _in 0.1 M cacodylate buffer. Tissues were dehydrated in a graded series of ethanol and embedded in Spurr's [38] plastic. Ultrathin sections were cut on a RMC MT-X microtome (Boeckeler Instruments) and collected on grids pretreated with formvar or parlodion. The sections were stained with alcoholic uranyl acetate and lead citrate [39] and examined at 120 kv with a FEI Tecnai 12 (formerly Philips) electron microscope. Semi-thin sections were stained with warming and a mixture of 0.5 g toluidine blue, 0.5 g sodium borate and 20 ml of methyl alcohol in 200 ml H_2_O.

## Results

Shown herein are typical examples of lamellar formation in successive developmental stages, but early book lung development is a continuum rather than a series of discrete steps related to the age of the individual. Within each scorpion there is a longitudinal gradation in the rate at which the book lungs develop, with the posteriormost pair (opisthosomal segment 7) the most advanced. In the early embryo, the limb buds of the third mesosomal segment gradually develop the features of pectines that temporarily overlap on the ventral surface of segment four [27,40-42]. The book lungs of this segment begin developing later than all those more posterior as the pectines separate from the ventral surface of the segment [27]. Additionally, within each book lung there is a gradation in the formation of lamellae; the most advanced lamellae are in the medial and central part of the book lung while the lateral edge has lamellae forming from newly aligned cells. Little or none of the book lung cuticle is shed as exuvium in the first molt [27], so most of the initial lamellae continue differentiation in the second instar.

### Epithelial proliferation, ingression and alignment

**Figure 1:**
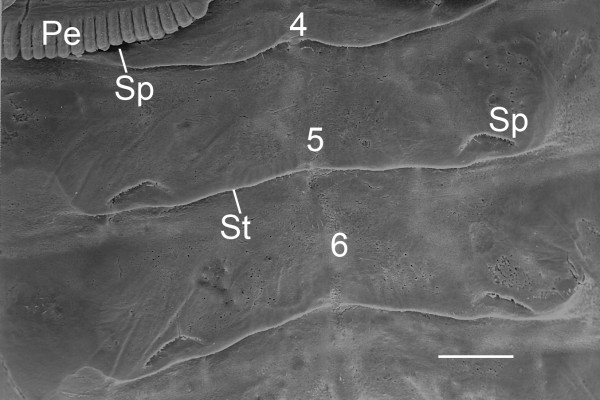
**Ventral view of embryo opisthosoma with spiracles (Sp) and small flap-like sternites (St) starting to form**. *Centruroides gracilis*, supine. SEM. The spiracles evident here are near the posterior margin of segments 4, 5 and 6. The spiracles on segment 4 are only partially visible because the pectines (Pe) from segment 3 still overlap on the ventral surface of segment 4. The spiracles are open with no closing mechanism until after the first molt (second instar). Scale, 200 μm.

As reported earlier [27,40-43], the limb buds on opisthosomal segments 4-7 disappear well before spiracles appear on the ventral surface. The entire segment 1 disappears while the limb buds on segment 2 become the genital operculum and those on segment 3 become the pectines. The spiracles are initially near the posterior margin of segments 4-7 (Figure 1). Also at the posterior segment margin, small flap-like primordial sternites appear about the same time as the spiracles (Figure 1). In later stages of the embryo and beyond, the sternites increase in size, and the spiracles are seen at a more anterior location, apparently as a result of differential growth of the tissues [27].

**Figure 2:**
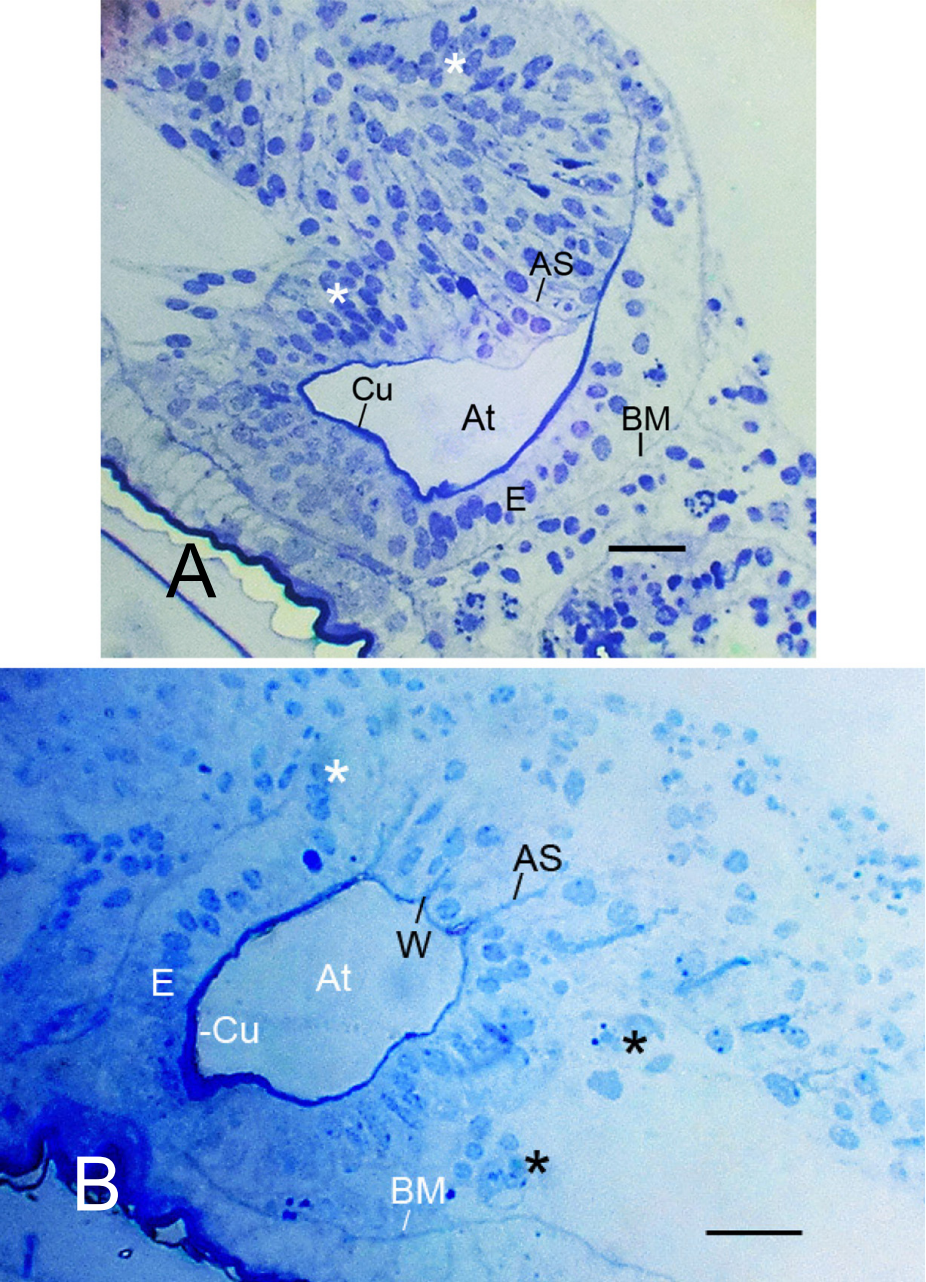
**Proliferation and migration of precursor book lung cells from the invaginated epithelium (E) of the atrium (At)**. Light micrographs (LM), ventral views, semi-thin sections. *Centruroides gracilis*. **A. **Newborn first instar. The air sacs (AS) in this book lung are at an early stage and barely discernible. Little or no widening is evident at the atrial origin of the air sacs. The cuticular wall (Cu) of the atrium is absent or very thin at the sites where air sacs are forming. The epithelial cells of the atrial wall are in a distinct layer where lamellae are not being formed; a basement membrane (BM) is present at the basal surface of these cells. The cells in the epithelial layer toward the left in the photo are more numerous as though proliferating near the site where air sacs are forming. On either side of the region of developing air sacs, the basement membrane is absent or disrupted and the precursor cells appear to be migrating inward (asterisks). **B. **Embryo book lung with some air sacs more advanced than those in Figure 2A. Inside the wall of the atrium, epithelial cells (E) of the hypodermis form a layer with a basement membrane (BM) at their basal surface. The cuticular wall (Cu) of the atrium is much thinner at the site where air sacs are forming. The primordial air sacs (AS) separate aligned precursor cells into double rows. Some cells (asterisks), not yet aligned into rows, appear to be dispersing inward from the atrial epithelium, and at these sites the basement membrane is disrupted or absent. Some widening (W) of the air sac entrance is evident at the atrial origin of two air sacs. In the lateral region (right) of the book lung, the developing air sacs are barely evident among cells not yet aligned. Scales, 20 μm.

**Figure 3:**
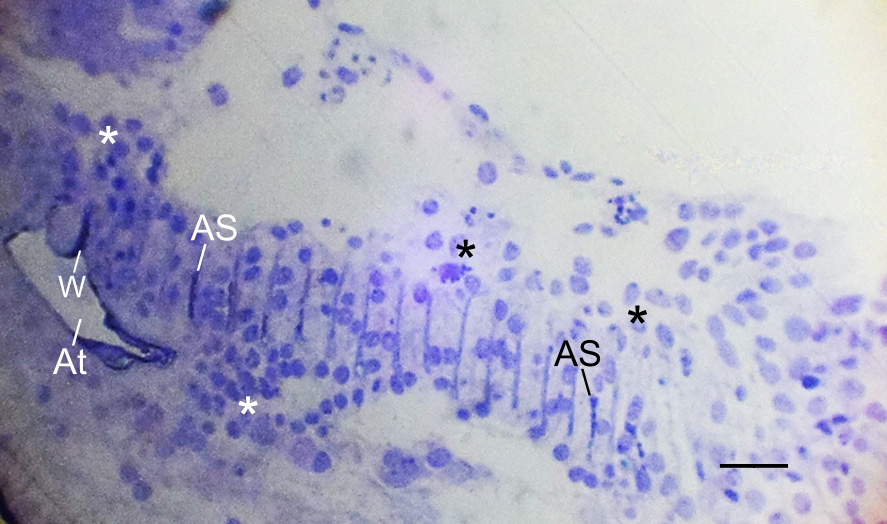
**Some air sacs (AS) with dense and opaque contents in a book lung more advanced than those in Figure 2A,B**. LM, ventral view, semi-thin section. *Centruroides gracilis*, newborn first instar. The portions of air sacs evident here are more prominent than in Figure 2A,B, and some air sacs (AS) are wider and more dense than the others because of the granular contents (see below). The air sacs are formed between double rows of cells. Some widening (W) of the air sac entrance is evident at the atrial origin of two air sacs. The asterisks indicate cells that are not yet aligned into rows or separated by primordial air sacs. Probably as a result of the proliferation and inward migration of cells, the atrium (At) no longer has an epithelial layer with basement membrane (compare with earlier stages in Fig. 2A,B). Scale, 20 μm.

The spiracles open into the atrium, a sac-like cavity formed by invagination of the external hypodermis at the spiracle site (Figures 2A, B and 3). The invaginated hypodermis consists of a layer of epithelial cells with apical-basal polarity, i.e., a thin cuticle at their apical surface and a basement membrane at their basal surface (Figure 2A, B). From this invaginated epithelium, the cells appear to proliferate and migrate inward (ingress) and arrange themselves in rows anterior to the atrium (Figures 2A, B and 3). As evident in the following figures, this alignment of the book lung precursor cells is the structural foundation for the regular pattern of page-like lamellae.

Figures 2A, B and 3 are book lung examples with increasing differentiation and development of the air sacs. As shown in these figures, the cuticle wall of the atrium becomes thinner where lamellae are forming, and the basement membrane is disrupted where cells are migrating inward. In Figure 3, an epithelial layer and basement membrane are no longer evident since the epithelial cells have apparently dispersed inward. The air sacs in Figure 3 are more prominent than those in Figure 2A, B, and some air sacs in Figure 3 are especially wide and dense as a result of their granular contents (described in more detail below).

**Figure 4:**
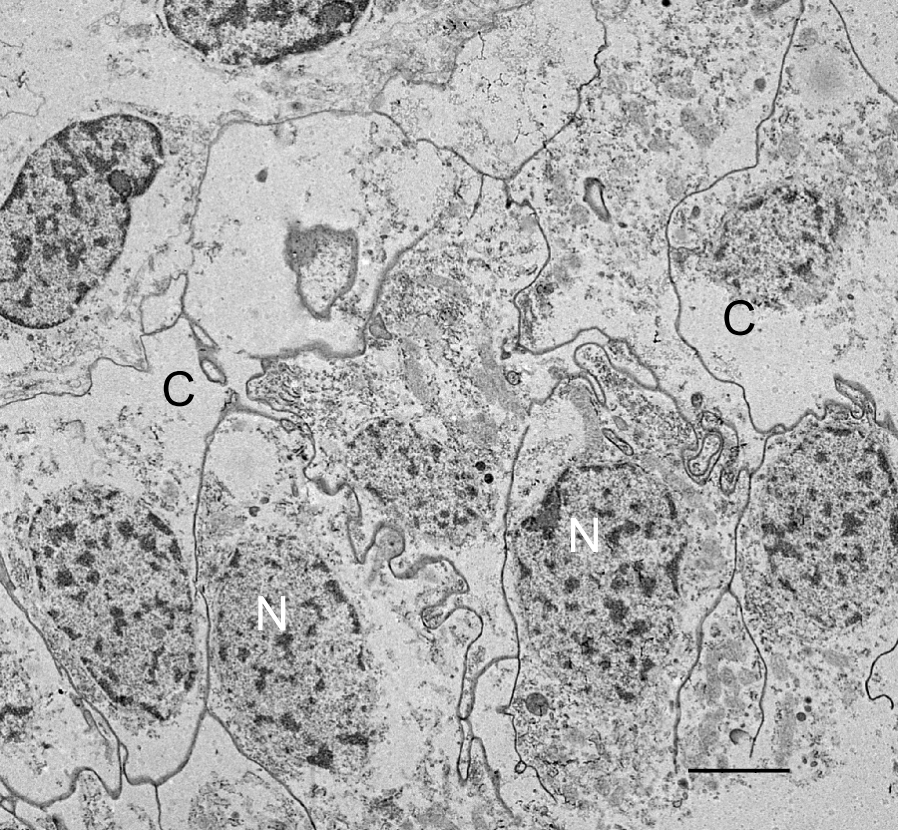
**Book lung precursor cells (C) not yet aligned anterior to the atrium**. *Centruroides gracilis*, embryo. TEM. The cells have irregular shape and many small particles, possibly ribosomes and granules. Primordial air sacs, hemolymph channels and apical-basal polarity are not yet evident among these cells. N, nucleus. Scale, 2 μm.

**Figure 5:**
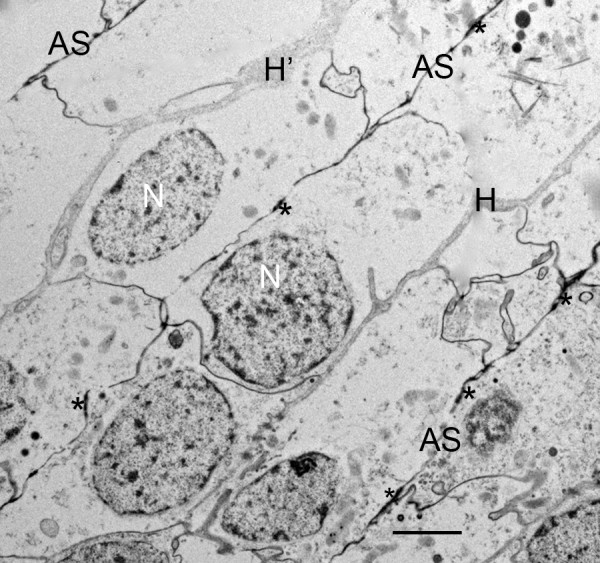
**Early stage in the development of air sacs (AS) among parallel, double rows of book lung precursor cells anterior to the atrium**. First instar, *Centruroides gracilis*. TEM. At this site somewhat inward from the atrium, the air sac channels begin with osmiophilic material (asterisks) that is apparently the result of merocrine secretion and/or enzyme action at the apical surface of the aligned cells. The basal surface of these cells is in contact with the primordial hemolymph channels (H), about 0.2 μm in width; one region (H') appears to be widening (~1.0 μm). Irregular wider regions like this gradually become more common. N, nucleus. Scale, 2 μm.

**Figure 6:**
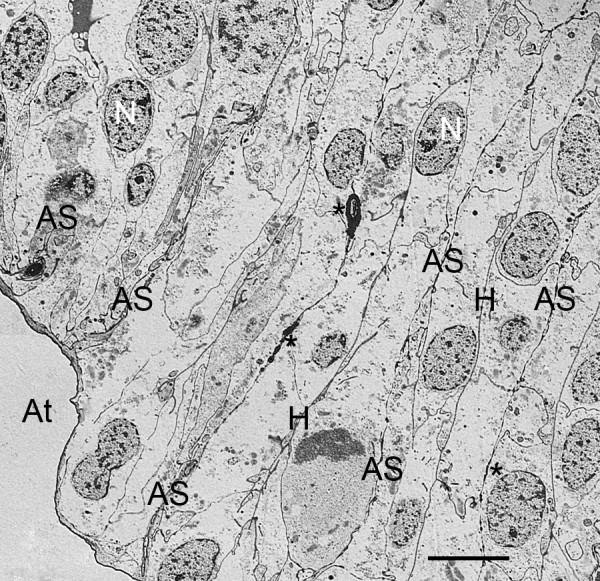
**Atrial lumen (At) with early indications of air sacs (AS) that are separated by double rows of aligned cells**. First instar, *Centruroides gracilis*. TEM. In some places closer to the atrium, the cells are releasing elongate fragments of cytoplasm as an early step in the development of the air sacs while farther inward there are only dense secretions (asterisks) that show the initial site of air sac formation (Fig. 5). No widening of the air sac entrance is evident at the sites where these early air sacs are forming. In these early stages, it is often difficult to identify where the air sac and hemolymph channels will be formed among the cells. N, nucleus; H, primordial hemolymph channel. Scale, 5 μm.

**Figure 7:**
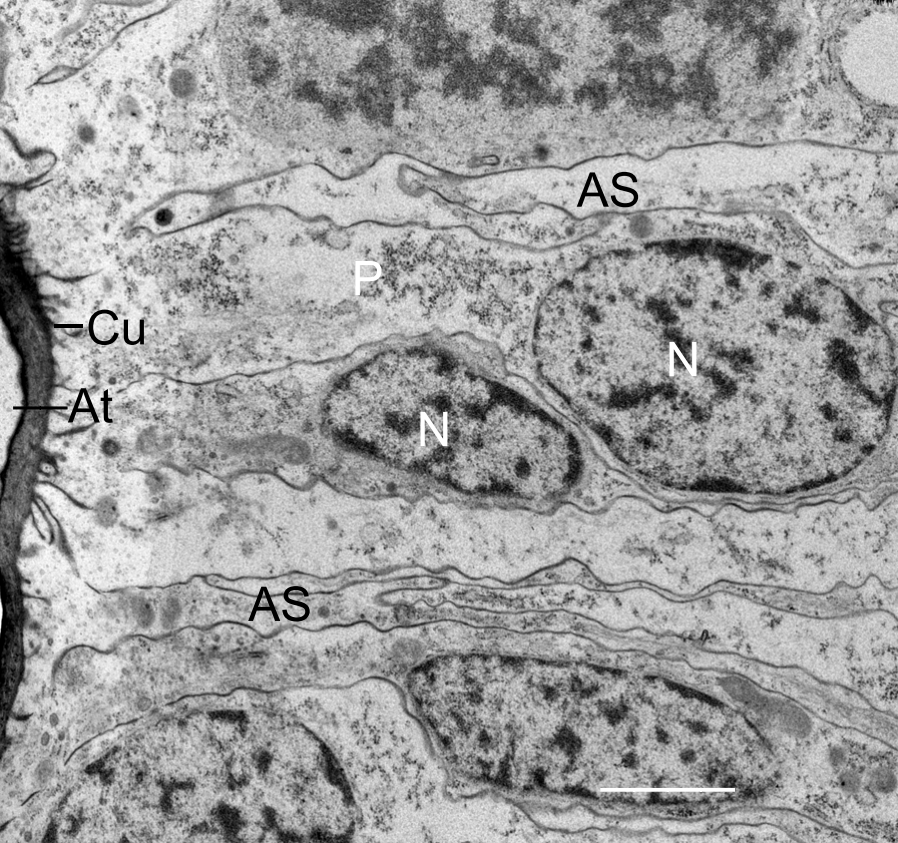
**Cell fragmentation as an early stage in the development of air sacs (AS) among double rows of book lung precursor cells**. No widening of the air sac entrance is evident at the origin of the air sacs in the cuticular wall (Cu) of the atrium (At), but two streams of elongate cell fragments separate a double row of cells from adjacent rows. The cells contain many small particles (P), possibly ribosomes and/or granules. The latter may contribute to the formation of air sac cuticle in later stages (e.g., Figs. 12, 13). N, nucleus. Scale, 2 μm.

**Figure 8:**
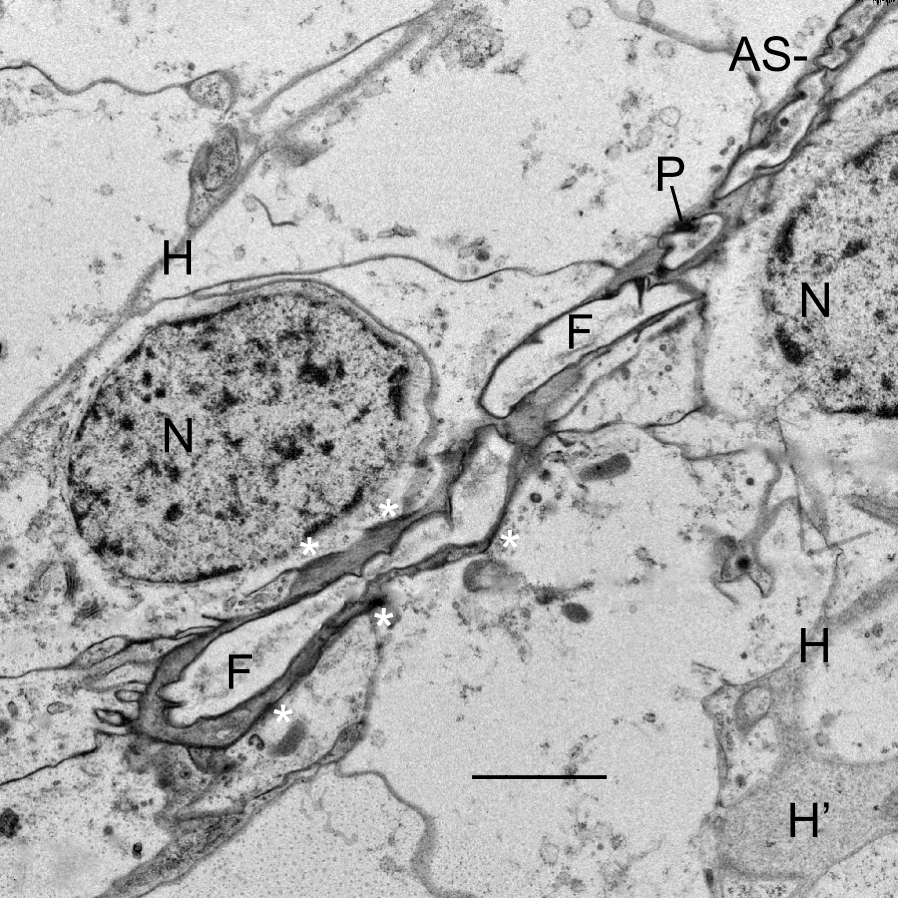
**Early air sac (AS) consisting of a single row of cell fragments (F) of similar width**. First instar, *Centruroides gracilis*. The aligned cells release fragments of cytoplasm from their apical surface (apocrine secretion). This results in a narrow channel (~1 μm wide) filled with cell fragments about the same width. The cells (N, nucleus) are commonly tapered toward the primordial channel as fragments are released. The cell fragments are initially enclosed in plasma membrane that gradually becomes thicker and more dense with presumptive cuticle (Figs. 9, 10, 12, 13). Small dense particles (P) at the periphery of the fragments are apparently formed from components inside the fragments and/or secreted from the apical surface of adjacent precursor cells. In addition to the cell fragments, the channel contains some osmiophilic material (probably fluid). The asterisks indicate short lengths of electron-opaque material that apparently results from merocrine secretion and/or enzyme action at the apical surface of the cells. The basal surface of the aligned cells is in contact with the primordial hemolymph channels (H) that are wider in some locations (H'). Scale, 2 μm.

**Figure 9:**
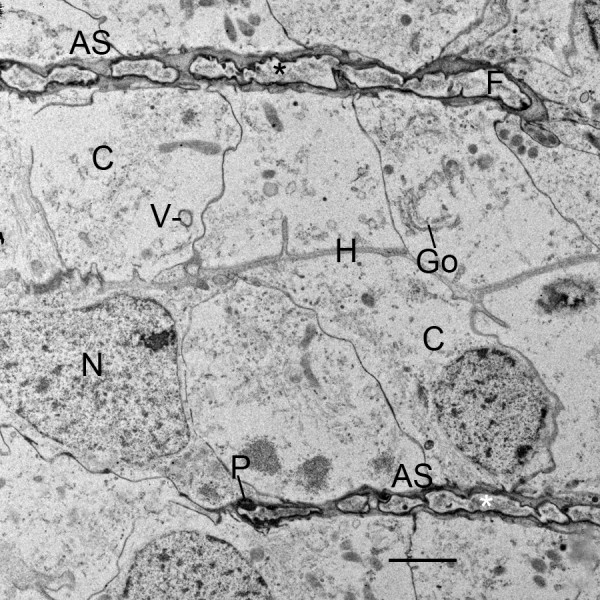
**Fusion and cuticularization of cell fragments (F) in developing air sacs (AS)**. First instar, *Centruroides gracilis*. TEM. The small fragments form an elongate row in a narrow channel that contains electron-opaque material, probably a fluid. The ends of the fragments make contact and appear to fuse together forming a more elongate structure. The initial plasma membrane of the cell fragments gradually becomes thicker and more dense as in cuticle formation. Small dense particles (P) at the periphery of the fragments are apparently formed from components inside the fragments and/or secreted from the apical surface of adjacent precursor cells (C). In some locations, these particles form a line (asterisks) as an early stage in development of a cuticular wall. The aligned precursor cells (N, nucleus) are commonly tapered toward the developing air sac, as though these cells have released or are about to release fragments. The cells have Golgi bodies (Go) and presumptive ribosomes, granules and vesicles (V) that are indicative of synthetic activity for cuticle formation (e.g., Figs. 12, 13, 14). H, primordial hemolymph channel. Scale, 2 μm.

**Figure 10:**
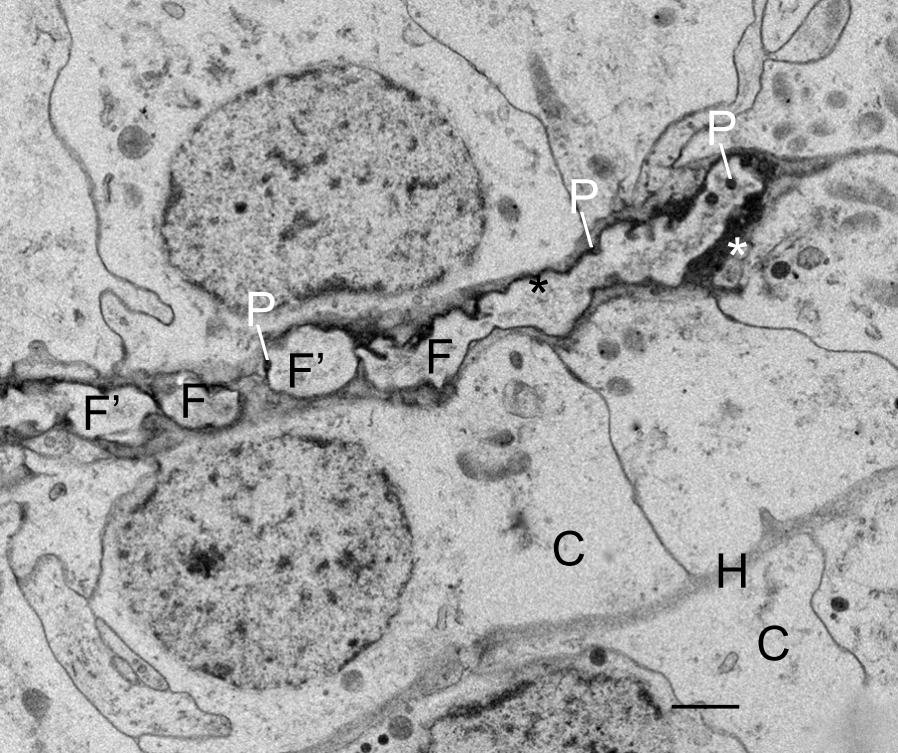
**Fusion and cuticularization of cell fragments in the primordial air sac**. First instar, *Centruroides gracilis*. TEM. Here at the anterior end of an elongating air sac, cell fragments in the air sac lumen appear to be fusing together (F, F') to form a continuous tube. The periphery of the fragments has varying thickness of electron-opaque material, an indication that cuticularization and formation of the cuticle wall of the air sac is more advanced in some places (black asterisk). Dense particles (P) are present inside and at the periphery of the fragments as though aggregating from components inside the fragment. The thick osmiophilic material near the tip of the air sac (white asterisk) may result from aggregation of particles at the periphery of the fragment and/or secretion from the adjacent cells. At this stage, most of the aligned precursor cells (C) appear to be intact except for releasing cell fragments at their apical surface (apocrine secretion). The primordial hemolymph channel (H) is still narrow (~0.3 μm) at this site. Scale, 1 μm.

**Figure 11:**
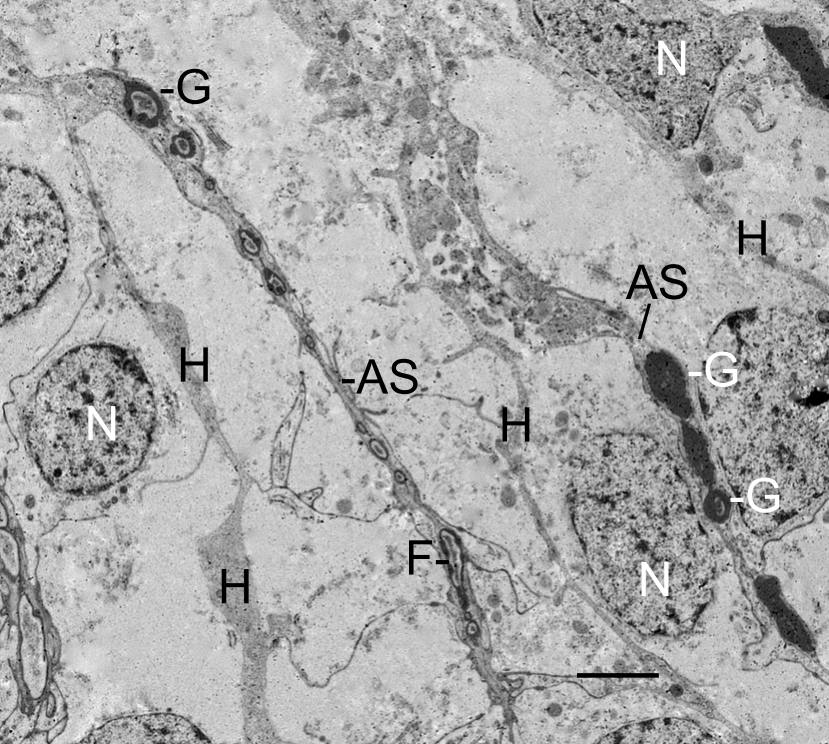
**Large, electron-opaque granules (G) in an early air sac (AS)**. Embryo, *Centruroides gracilis*. TEM. Cells with such granules as much as ~1 μm dia. are sometimes seen in the developing book lung tissue of embryos and first and second instars. These cells contribute cytoplasmic granular portions to the developing air sacs (Fig. 3). The result as shown here is a linear series of these granules that gradually become more lucent in their center. As shown in Figures 8, 9 and 10, small dense particles aggregate at the periphery of the cell fragments (F) as though an early stage in the formation of a continuous cuticular wall. The pale center of some large granules (G) suggests a similar movement of material toward the periphery where the separate fragments can fuse and form continuous cuticular walls on either side of the air channel (Figs. 12, 13, 14, 15, 16, 17). H, primordial hemolymph channel; N, nucleus. Scale, 2 um.

**Figure 12:**
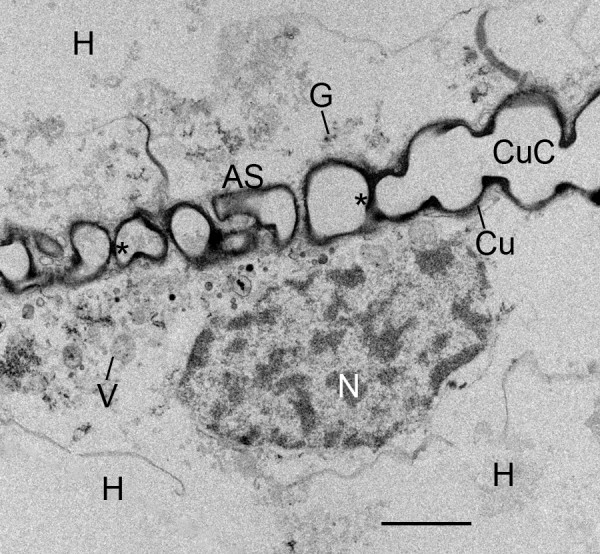
**Formation of space holders (asterisks) and longer lengths of air sacs as a result of fusion and cuticularization of former cell fragments, now cuticle-enclosed components (CuC) of the air sac**. First instar, *Centruroides gracilis*. TEM. Compared with earlier stages (Figs. 8, 9, 10, 11), the outer wall of these components is now relatively thick (~0.1-0.2 μm) and dense. The walls of these components appear to fuse (asterisk) and form a bridging trabecula (space holder) that helps hold the developing cuticular walls in place (Figs. 13, 15, 16, 17). Many of the aligned precursor cells like this one (N, nucleus) release their contents and deteriorate (holocrine secretion) between the developing air sacs, thereby resulting in hemolymph channels (H) with increasing width and space for passage of fluid. Granules (G) and vesicles (V) from these cells appear to increase the thickness of the cuticular walls (Cu, Fig. 13) of the developing air sacs. Scale, 2 μm.

**Figure 13:**
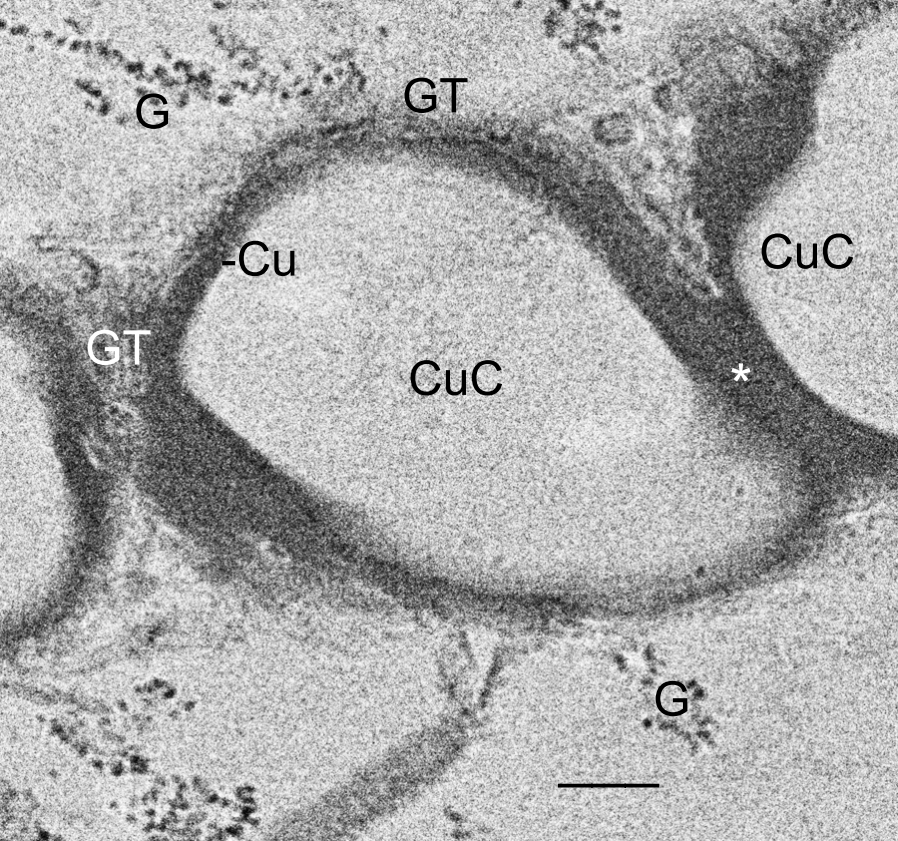
**Formation of space holders (asterisk) and longer lengths of air sac as a result of fusion and cuticularization of the former cell fragments, now cuticle- enclosed components (CuC) of the air sac**. First instar, *Centruroides gracilis*. TEM. Small dense granules (G, 20-30 nm dia.) from the adjacent aligned cells (e.g., Fig. 12) appear to fuse with the developing cuticular wall (Cu), giving it a granular texture (GT) at some sites where the wall is forming. This process appears to be similar to cuticularization observed with SEM [1,27]. The fused cuticle from adjacent components (CuC) may continue as space holders (bridging trabeculae) evident in more advanced air sacs (e.g., Figs. 15, 16, 17). Scale, 200 nm.

**Figure 14:**
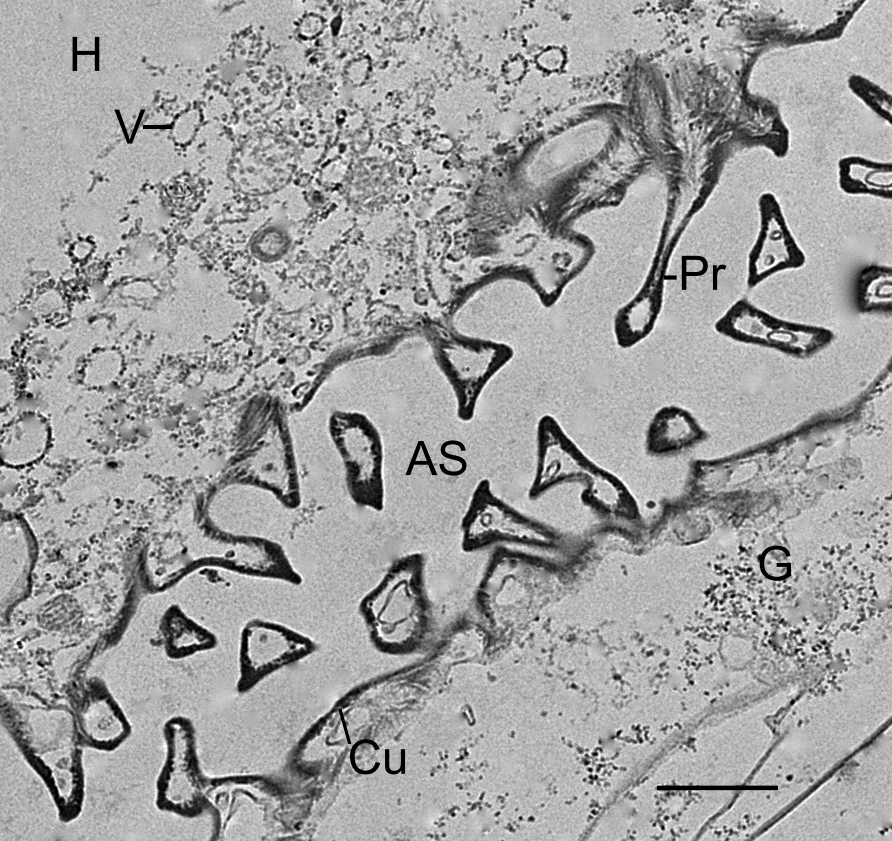
**Formation of space holders by extension of cell processes (Pr) into the lumen of the developing air sacs (AS)**. First instar, *Centruroides gracilis*. TEM. Cells adjacent to the air sac wall contain many vesicles (V) and granules (G) that are apparently components for the thickening cuticular wall (Cu). The cell processes (trabeculae) also contain vesicles and granules so apparently the protrusions can gradually enlarge and extend across the lumen, forming wall-to-wall (bridging) space holders (e.g., Figs. 15, 16, 17). H, primordial hemolymph channel. Scale, 1 μm.

Figures 2A, B and 3 have regions where the precursor cells are not yet organized into double rows separated by developing air sacs. Some cells at this stage are shown in the electron micrograph of Figure 4. These cells are irregular in shape and show no indication of the apical-basal polarity that is prominent in subsequent stages (Figures 5, 6, 7, 8, 9, 10, 11). The cells have many fine particles, probably ribosomes and granules; the latter may eventually become part of the cuticular walls of the air sacs (Figures 12, 13, 14).

### Apical-basal polarity, secretion

Beginning at the atrium, the aligned precursor cells soon begin to show indications of the polarity that is common among epithelial cells, i.e., secretion at the apical surface while the basal surface is in contact with hemolymph where nutrient transport can occur [44]. This difference in activity between the apical and basal surfaces of the aligned cells results in a regular pattern of developing air sacs among double rows of cells (Figures 2A, B and 3, 6, 7, 8, 9, 10, 11). Since the precursor cells are in parallel rows, their secreted product (i.e., primordial air sacs) are also in parallel rows. Air sacs separated by double rows of cells were observed in early light microscopic studies of book lung development in spider embryos [8,9,18,20,21,23,24,45]. Some of these authors also observed slight widening at the atrial entrance of a few air sacs. As described in Background, this widening was hypothesized to be evidence of hypodermal invagination (infolding) as the basis for the cell alignment anterior to the atrium.

In this scorpion investigation, some widening of the air sac entrance was seen for some more advanced air sacs (Figures 2B, 3), but not in the initial stages of air sac formation (Figures 2A, 6, 7) as would be expected if infolding of the atrial wall hypodermis had resulted in the cell alignment for the air sacs. As noted in Background, some presumptive folds in the atrial wall were interpreted as evidence for atrial wall invagination in the formation of book lung lamellae in scorpion embryos, although a relationship between folds and lamellae was not shown [19,22]. The structures diagramed as folds may have been small widenings of the air sac entrance as observed in the book lungs of spider embryos.

In the initial stages of air sac formation, a thin line of osmiophilic material suggests merocrine secretion from the apical surface of the aligned cells (Figures 5, 6), and the release of apical portions (apocrine secretion) of these cells (Figures 6, 7, 8) forms an elongate channel of cell fragments (Figures 8, 9, 10, 11). In later stages, entire cells are disrupted (holocrine secretion, Figure 12), increasing space for hemolymph at the base of the remaining cells and contributing components to the cuticular walls of the air channel (Figures 12, 13).

Figures 6, 7 and 8 show early stages of cell fragmentation from the apical surface of the aligned cells. This process starts near the atrium, and continues anteriorly among the cell rows. It is sometimes difficult to recognize the location of the future air sacs and hemolymph channels among the disrupted cell components, and there is no widening of the air sac entrance at the atrial wall where the cell fragmentation is occurring (Figures 2A, 6, 7). The cell fragments are surrounded by a plasma membrane, and the fragments contain vesicles and granules (Figures 6, 7, 8) as in the cells of origin. In addition, the fragments are in a narrow and elongate lumen that is somewhat dense (Figures 8, 9, 10, 11) as though a fluid is present with material secreted from the apical surface of the adjacent cells.

### Fusion of cell fragments, cuticle formation

Within the primordial air channels, the cell fragments appear to fuse together to form more elongate strands of membrane-bound cytoplasm (Figures 8, 9, 10, 11). The fragments and secretions from the aligned cells apparently contain enzymes and molecular components for cuticle since electron-opaque particles are formed within the cell fragments. The dense particles appear especially at the outer membrane of the cell fragments (Figures 8, 9, 10, 11) where they coalesce in the formation of a continuous cuticular wall (Figures 9, 10). There are regions of increased density and thickness of the outer covering of the conjoined cell fragments (Figures 9, 10). Thin lines of electron-opaque material (presumptive merocrine secretion) can also be seen at the apical surface of the cells adjacent to the developing air sacs (Figures 8, 9, 10). The initial cuticular wall of the developing air sac is apparently produced by formation and coalescence of dense, electron-opaque material within the cell fragments and secreted at the apical surface of the precursor cells.

In these early stages of book lung development, cells with large (~1 μm dia.) electron-opaque granules are sometimes seen among the other precursor cells. Examples were seen where these cells release fragments with large granules that become part of the developing air sac. Once inside the primordial air sac, these granules make the air sac somewhat wider and more dense than the other air sacs without these granules (Figures 3, 11). The center of the granules becomes lucent (Figure 11) as though the material is removed and/or migrating toward the periphery of the granule where fusion of the fragments and coalescence of the dense material can result in a continuous cuticular wall. This process may be similar to the coalescence of small particles at the periphery of cell fragments in the primordial air sacs (Figures 8, 9, 10, 11).

As shown in Figures 8, 9, 10 and 11, the outer wall of the air sac fragments gradually becomes dense and thicker, so that the former cell fragments enclosed in plasma membrane are now cuticle-enclosed air sac components, labeled as CuC in Figures 12 and 13. The cell fragments and more advanced cuticle-enclosed components appear to fuse and form a continuous, elongate structure (Figures 9, 10, 11, 12). Some cells adjacent to the air sacs are disrupted as they release granules and vesicles that appear to increase the thickness of the cuticle walls (Figure 12). The disruption leaves cell debris and additional space for fluid in the primordial hemolymph channels. Granules and vesicles from the deteriorating cells are often seen in contact with the inside and outside of the cuticular walls as though merging with the wall and/or releasing components that become part of the wall (Figures 13, 14). This process of cuticle formation in scorpion embryos was also observed in earlier studies with SEM [27] and TEM [46].

**Figure 15:**
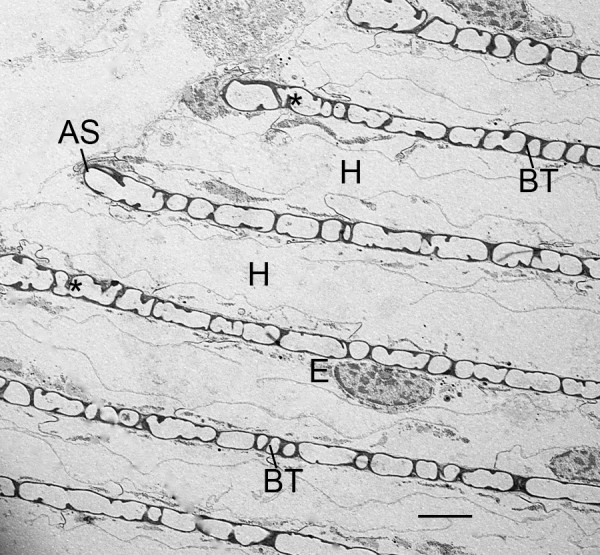
**Regular pattern of air sacs (AS) and hemolymph channels (H) that result from developmental processes like those described herein**. First instar, *Centruroides gracilis*. TEM. There is a thin epithelial layer (E) attached to the hemolymph surface of the air sacs. The hemolymph channels (H) still have some cell debris, but are nearly open for passage of fluid. Evident are many bridging trabeculae (BT) that extend between the air sac walls. These space holders apparently result from the fusion of smaller cuticle-enclosed compartments (Figs. 12, 13) or extension of cell processes (Fig. 14) and trabeculae (asterisks) into the lumen of the developing air sacs. The air sacs here are ~1.5 μm wide while the hemolymph channels are 5-7 μm wide. Scale, 5 μm.

**Figure 16:**
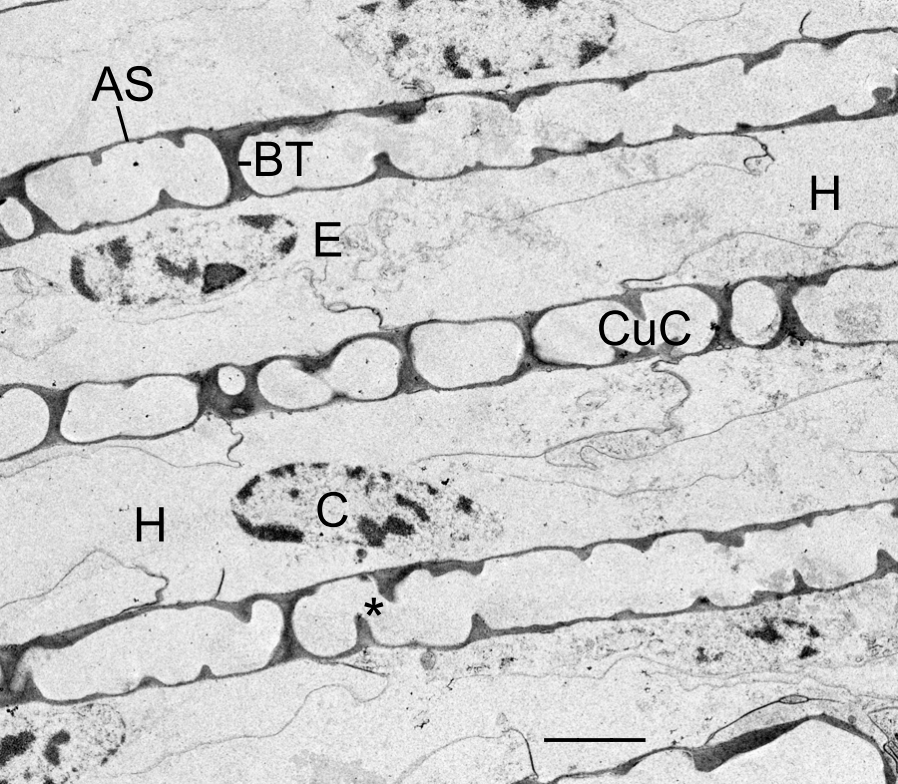
**A regular pattern of air sacs (AS) and hemolymph channels (H) in the book lung of a newly molted second instar**. *Centruroides gracilis*. TEM. The first instars molt while on their mother's back, but the development of the book lungs continues without interruption. Bridging trabeculae (BT) appear to be formed by fusion of small cuticularized components (CuC, Figs. 12, 13) or by cuticularization of cell processes that extend into the lumen of the air sac (asterisk, Fig. 14). One cell (C) shown here in the hemolymph channel (H) is ruptured and deteriorating while others may remain intact and part of the epithelial layer (E) attached to the hemolymph surface of the air sacs. The hemolymph channels at this location are 3-4 μm wide and have a substantial amount of cell debris. The air channels are 1.5-2.0 μm wide and are open for the passage of air except for the trabeculae. Scale, 2 μm.

**Figure 17:**
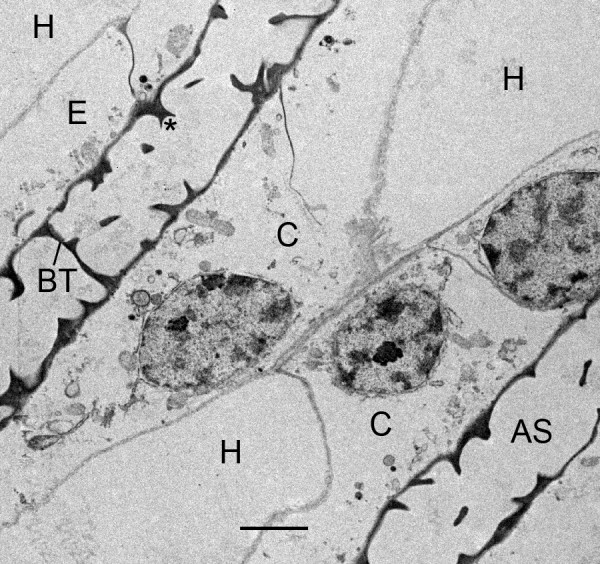
**Presumptive primordial space holder across the hemolymph channel (H) of a second instar book lung**. *Centruroides gracilis*. TEM. Many cells deteriorate in the primordial hemolymph channel (Figs. 12, 16), resulting in space for passage of hemolymph. The two intact cells (C) shown here make contact across the channel lumen. These may be precursor pillar cells for the pillar type space holders that earlier workers report are a common feature in the book lung hemolymph channels of adult scorpions and spiders [30,45,47-50]. AS, air sac; BT, bridging trabeculae; asterisk, non-bridging trabecula. Scale, 2 μm.

As shown in Figures 2A, B, 3, 4, 5, 6, 7, 8, 9, 10, 11 and 12, the precursor cells (~ 3-8 μm wide) are much larger than the early air sacs. The width of the air sacs is determined by the amount and size of the material released from the apical surface of the aligned cells. The latter apparently release similar quantities since each of the resulting channels has a fairly uniform width throughout their length. In these early stages of development, the channel width varies from a dense line (~ 0.1 μm, Figures 5, 6) to an elongate row of fragments 1-2 μm in width (Figures 8, 9, 10, 11). With the loss of cells and likely exchange of materials across the basal surface of the aligned cells, the examples of hemolymph channels herein increase in width from ~ 0.3 μm (Figures 8, 9, 10, 11) to ~ 6 μm (Figures 15, 16, 17).

### Formation of space holders (trabeculae)

Within the air channel lumen, trabeculae may be formed by fusion of the cuticularized walls of the former cell fragments (Figures 12, 13). Cells attached to the developing cuticle walls of the channel can also extend processes into the lumen of the air sac (Figure 14). These processes often contain granules and vesicles that may become part of the cuticle. Apparently, the cellular processes continue elongation into the lumen, make contact with the opposite wall and become cuticularized. Trabecular formation results in many space holders that help hold the cuticle walls in place and prevent their collapse and blockage of air flow in the narrow and elongate channels. Space holders that connect with both walls of the air channel (bridging trabeculae, cross bridges) is the only type seen in the present investigation of the early stages of book lung development, although numerous other types of space holders are found in adult scorpions [30]. In this investigation, there are many examples of trabeculae that do not extend all the way to the opposite wall (Figures 14, 15, 16, 17), possibly because the space holder is incomplete or the entire bridging trabecula was not evident in the section.

As shown in Figure 15 (first instar), a regular pattern of lamellae results from the somewhat disorderly process of cell secretion and disruption described above. Trabeculae that partially and completely span the width of the air channels are abundant in the example of Figure 15. The air sacs contain little cell debris and are nearly open for passage of air except for the slender trabeculae. A thin layer of epithelial cells is attached to the hemolymph surface of the air sacs. These cells form a hypodermis in position to produce page-like replacement cuticle in later molts.

Some of the first instars examined in this investigation were thought to be close to their first molt, but in the sections there was no indication that lamellar epithelial cells were secreting new cuticle walls as preparation for the first molt. As shown in an example of first-molt exuvium [27], some replacement of lamellar cuticle may be needed only in the distal parts of the posteriormost booklungs.

### Hemolymph channels

Figure 16 shows an example of book lung lamellae from a second instar within 24 hours after the first molt on the mother's back. The lamellae appear to be at a similar stage of development as those in the advanced first instar of Figure 15. Some cells attached to the hemolymph surface of the air sac wall appear to be deteriorating while others are intact and may continue to replace cuticle shed in later molts [27]. The air sacs have some bridging trabeculae that were probably formed by fusion of small air sac components with larger ones. The hemolymph channel contains much cell debris, apparently from the double row of precursor cells that were previously aligned between the developing air sacs. The width of the hemolymph channel here is ~ 4 μm, i.e., the approximate width of two epithelial cells side-by-side.

Figure 17 provides further evidence that the width of the hemolymph channel may be related to the width of two precursor cells. While many precursor cells deteriorate (Figures 12, 16), others remain as part of a thin epithelium attached to the air sac wall (Figures 15, 16) [27]. The two cells in Figure 17 have made contact across the hemolymph space. They may be primordial pillar cells described as the space holders for the hemolymph channels of adult book lungs in scorpions and spiders [30,45,47-50].

Cells with large (~1 μm dia.), electron-opaque granules did not appear to be abundant among the book lung tissue in the early stages of this investigation. Short lengths of air sacs contain these granules (Figures 3, 11), but most primordial air sacs are formed from secreted cell fragments that lack such granules (Figures 2A, B and 3, 8, 9, 10). Some granular cells were seen in the hemolymph channels of the book lungs of second instars. These are probably hemolymph-borne granulocytes; their presence is expected as the book lungs become functional for the active and foraging second instars. There may be different types of these granular cells, although their morphology is similar in the stages examined herein. Granulocytes have been described in scorpion hemolymph [51,52], and an investigation with TEM showed the opisthosomal lateral lymphoid and supraneural glands are likely sources of such cells  [52].

## Discussion

### Epithelial cell invagination, proliferation, ingression and alignment

As reviewed by Fristrom [44], epithelial cells are characterized by a stable apical-basal polarity that is maintained throughout development, and their lateral surfaces have intercellular junctions that connect them to adjacent cells. In recent years, there is much research on the molecular basis for the polarity in epithelial cells [53-58] as well as their intercellular junctions [44,55,57,59-61]. Tyler [62] concludes that epithelial cells appear first in the Eumetazoan blastula and are a prominent feature in subsequent adult tissues. The characteristic apical-basal polarity of these cells, a critical factor in the present investigation, may also have arisen very early in stem metazoans.

Epithelial cells commonly exist in sheets and provide protective coverings and barriers for the external surface and for tubes, vessels and channels inside the animal. They are commonly in a single layer with a basement membrane secreted from their basal surface. Fristrom [44] notes that invertebrates sometimes have epithelial cells without a basement membrane, i.e., the cell basal surfaces are directly in contact with hemolymph as in Figures 15, 16 and 17. In arthropods, including scorpions, an important and typical example of epithelium is the hypodermal layer that secretes and replaces the cuticle and forms many external and internal structures [1,63-69].

Epithelial cells undergo many different morphological changes during development; invagination of a specific region of the epithelium (Figures 1, 2A, B) is a common example [44,55]. The invagination often results from constriction of the apical ends of the cells [70,71] with the apical ends lining the concave surface, e.g., the atrial cavity of book lungs (Figure 2A, B). In scorpion embryos, the atrial wall initially consists of a layer of hypodermal cells with a basement membrane at their basal surface (Figure 2A, B).

As explained in Background, the invagination or infolding of the atrial epithelium for each lamella of arachnid book lungs was proposed to be a continuation of the small amount of invagination that may occur along with outgrowth (evagination) in the formation of book gills of horseshoe crabs [7]. Slight widening at the atrial origin of some spider air sacs was hypothesized to be an indication of atrial wall infolding to provide the aligned precursor cells for each air sac [8,9,18-21]. In other light microscopic studies of spider embryos [23,24], the authors noted some air sac widening at the atrial wall, but this was not considered a necessary or characteristic feature of air sac development.

In the present investigation, widening of the air sac entrance was seen at the atrial origin of some advanced lamellae (Figures 2B, 3) but not in the earliest stages of lamellar formation (Figures 2A, 6, 7). Without folds or invaginations from the atrial wall, the epithelial cells in scorpion embryos appear to proliferate, ingress (migrate inward) and organize themselves into parallel rows anterior to the atrium (Figures 2A, B and 3, 5). Epithelial ingression is a common developmental process involving breakage of cell-cell adhesions, migration out of the epithelial layer and formation of new cell-cell adhesions [44,55,72]. As in Figures 2A, B and 3, there is typically a loss of the basement membrane at the site of migration from the epithelium [44].

Among vertebrates and invertebrates, the ability to form a single row or layer of cells is a common feature of epithelial morphogenesis [44,72-74]. Often a single row, layer, cavity, tube or branching tube (e.g., tracheae, bronchi, blood vessels) is formed, while in book lungs the precursor cells organize themselves into multiple, nonbranching rows parallel to each other. The book lungs thus have much surface area for gas exchange without the added complexity of epithelial branching. A comparable nonbranching tracheal structure is the sieve trachea of spiders (Mysmenidae) such as *Calodipoena incredula *and *Mysmenella samoensis *(formerly *Mysmena incredula *and *M. samoensis *respectively [37,75]. Here, clusters of parallel, tubular tracheae extend forward from a common atrium.

### Epithelial cell polarity, secretion and lamellar formation

Initially in their ingression from the atrial epithelial layer, the book lung precursor cells have irregular shapes and show no sign of polarity (Figures 2A, B and 3, 4). Like epithelial-derived cells elsewhere (e.g., exocrine glands, tracheae, bronchi) [44], the aligned cells anterior to the atrium soon show evidence of atrial-basal polarity, i.e., secretion from the apical surface (Figures 5, 6, 7, 8, 9, 10, 11) while the basal surface is in contact with the nutrient source (hemolymph). The enzymes and molecular components for the exoskeleton are secreted from the apical surface of the parent hypodermal cells [1,29,63-69], and the material for the cuticle of the air sacs is secreted from the apical surfaces of the aligned book lung precursor cells (Figures 5, 6, 7, 8, 9, 10, 11).

As the aligned cells begin to release material from their apical surface, their atrial-basal polarity results in double rows of cells separated by primordial air sacs (Figures 5, 6, 7, 8, 9, 10, 11). The apical secretion produces the air sacs while the hemolymph channels form at the opposed bases of the aligned cells. The release of electron-opaque material (Figures 5, 6; presumptive merocrine secretion) and cell fragments (Figures 6, 7, 8, 9, 10, 11; apocrine secretion) is characteristic of the early stages of lamellar formation. In more advanced stages, entire cells are disrupted, releasing granules and vesicles (holocrine secretion) that apparently contribute to the thickening cuticular walls of the air sacs (Figures 12, 13, 14).

While secretion from the apical surface of epithelial cells is a common feature [44,76-79], the cellular process of air sac formation is distinctive as described here for scorpions. The released cell fragments are similar in width among the aligned cells (Figures 8, 9, 10, 11), and the fragments fuse together to form an elongate channel (Figures 8, 9, 10, 11, 12, 13, 15, 16) with gradually thickening cuticular walls. A similar process of lamellar formation may occur in other arachnids as suggested from recent [50] and early studies of book lung lamellae in adults [8,9,21,23,24,45].

For scorpion book lung lamellae, the cell fragments are initially enclosed in a typical plasma membrane (Figures 6, 7, 8). Later, the covering of the fragments appears thicker and more dense as though cuticle is forming. The electron-opaque structures in the micrographs give some indication of how cuticularization occurs, but the process is uncertain. There are electron-opaque particles at the periphery of the fragments as though material in the fragment has begun to aggregate and/or form cuticle by enzyme action (Figures 9, 10, 11). These peripheral particles and the dense and thickened fragment walls may also be all or partially formed from the contents of fluid in the narrow and elongate lumen that contains the cell fragments (Figures 8, 9, 10, 11). Whatever the process, the cuticular wall of the air sac components is 50-200 nm thick in Figures 12 and 13.

As described above, an important feature of cuticularization is the apparent aggregation and/or formation of cuticle at the periphery of the cell fragments where fusion of the fragments can result in a continuous cuticle wall. The cells with large, electron-opaque granules may add larger quantities of cuticle primordia to the developing air sacs (Figures 3, 11), i.e., the peripheral coalescence of the presumptive cuticle material (Figure 11) helps form a continuous wall from the fused cell fragments in the developing air sac.

In an earlier study with SEM, entire small cells appeared to become aligned and enclosed within the cuticular walls of the developing air sacs [27], while in the present investigation with TEM the air sacs are formed from cell fragments of larger aligned cells. This difference in cell contribution to lamellar formation results from a difference in the stage of development and the location within the book lung where the developmental process is viewed with the two different types of microscopes. Most of the sections in the present study were taken at an early stage and parallel to the aligned cells and developing lamellae. The earlier SEM study [27] showed the dorsal surfaces of lamellae at successive stages of development.

In the present investigation, the lamellar precursor cells are 3-8 μm in width (Figures 2A, B and 3, 4, 5, 6, 7, 8, 9, 10, 11, 12, 14, 17) while the primodial air sacs are much smaller, increasing in width from ~0.5 μm (Figures 5, 6) to 2-3 μm (Figures 9, 10, 11, 13, 14, 15, 16, 17). Cell fragments rather than entire cells become part of the early air sacs. In the TEM sections observed herein, small cells 2 × 4 μm were sometimes seen among the larger ones, but they were not seen to become enclosed within the developing air sacs. In the early stages, each row of cell fragments is ~1 μm dia. or less, so even the smallest cells are too large to become part of the early air sac.

In the earlier study with SEM [27], it appears that the lamellae are increased in length, height and number by addition of material at (respectively) the anterior, dorsal and lateral edges of the book lung. A porous membrane overlies the dorsal surface of the book lungs [80,81], and this was removed so the SEM could be used to view the developing lamellae from the dorsal aspect. The growth regions of the book lung, thus exposed, have many small cells 2 × 4 μm, and these small cells appear to become aligned and surrounded by cuticle as they become part of the vertical growth of more advanced and wider (2-3 μm) lamellae. Openings in the lamellae showed cells and/or large granules deteriorating and apparently providing cuticle components [27].

### Development of space holders

As pointed out by Farley [1,27] book lung space holders are described in numerous earlier papers; most helpful is the comprehensive treatise by Kamenz and Prendini [30]. The present investigation is the first to provide ultrastructural information about the formation of book lung space holders in the early stages of development.

In the book lungs of adult scorpions, the proximal one-third to one-fifth of the air sac lamellae have bridging trabeculae while the remaining distal portion has other types of nonbridging trabeculae that are firmly attached to only one wall of the air sac [30]. As reported in that study, for adult *Centruroides gracilis *the distal part of each lamella has a reticulate network of cuticular veins that serve as nonbridging space holders while bridging trabeculae are present in the smaller proximal part of the lamellae. A similar lamellar pattern of bridging and nonbridging trabeculae is present in adult spiders. For spider lamellae, Purcell [8] concluded that the proximal portion with bridging trabeculae is formed by new growth during the preceding intermolt period, while the distal part of the lamellae has nonbridging trabeculae attached to only one wall because there is temporary obstruction by the lamellar exuvium during the molt.

The replacement of book lung cuticle has not yet been studied in advanced scorpion instars, but present results suggest that Purcell's [8] hypothesis may be applicable. The lamellae in adult *Centruroides gracilis *have different proximal and distal trabeculae [30] as in spiders, but in the present investigation with embryos and early instars, only partial or complete bridging trabeculae were seen (Figures 15, 16, 17). These trabeculae probably provide much stability for air sac walls in the early stages of development when lamellar exuvia and replacement cuticle are not yet factors.

The bridging trabeculae in scorpion embryos and first instars may be formed by the fusion of smaller components in the developing air channels (Figures 12, 13). In more advanced stages, the lamellae have regions where it appears that air sac components fused with others, and bridging trabeculae were formed in the process (Figures 15, 16). In Figures 15, 16 and 17, partial trabeculae are common, but it is not clear if they result from sectioning of a bridging trabecula and/or incomplete wall-to-wall growth.

Figure 14 shows the development of trabeculae by extension of cell processes into the lumen of the developing air sac. The cell processes and adjacent cellular debris contain vesicles and granules that may contribute to cuticularization and extension of the processes to the opposing wall. Space holders are being formed here from processes that extend inward from both walls of the air sac rather than one wall as occurs for nonbridging trabeculae [30]. In the opisthosomal appendages and book gills of the horseshoe crab, trabeculae are formed by cuticularization of cell processes from the adjacent epithelial (hypodermal) cells [1].

Space holders are much less common in the hemolymph channels as compared with the many trabeculae in the air sacs [27]. In adult horseshoe crabs and adult scorpions and spiders, the hemolymph space holders are mainly cellular pillars, i.e., conjoined extensions of pillar cells from opposed cuticle walls [30,47-50]. Examples that may be the initial formation of cellular pillars are provided in Figure 17 and earlier investigations [1,27]. The two conjoined cells of Figure 17 span the hemolymph channel and may be retained as pillar cells, while most of the cells in the primordial hemolymph channel become part of the epithelium attached to the air sac wall (Figures 15, 16, 17) or they deteriorate and thereby leave space for hemolymph (Figures 12, 16).

### Comparison of book gill and book lung development

In spider embryos, the spiracles, atrium and primordial air sacs develop posterior to opisthosomal limb buds; book gills are formed from the posterior surface of opisthosomal limb buds of the horseshoe crab [1,6-9,18,20,21,45]. In scorpion embryos, the limb buds have completely disappeared, except for those that become the genital operculum and pectines, before spiracles are evident on the ventral surface of opisthosomal segments 4-7 (Figure 1) [27,40-43]. This does not negate the possibility of book gill/book lung homology, since embryo development is often modified for the benefit of the embryo [[82], p. 128], as demonstrated by the substantial developmental adaptations for oral feeding in the more derived scorpion families [65,83-86].

As described above, infolding or invagination of the atrial epithelium was not evident herein as a source of the precursor cells for each lamella. Rather, there is ingression of cells from the atrial epithelium (Figures 2A, B and 3), and their subsequent alignment, apical-basal polarity and secretion result in the highly ordered lamellae of the book lungs (Figures 15, 16). These developmental cell processes produce an arrangement of cells and lamellae that would have occurred if infolding of the atrial epithelium had been the source of the precursor cells for each lamella. This raises the possibility that such infolding was an ancestral condition that initiated lamellar formation but is no longer evident in extant scorpions. This hypothesis, however, requires replacement of epithelial infolding with ingression in the evolutionary history of the book lungs.

In the horseshoe crab, the outgrowth (evagination) of the book gill lamellae from the posterior surface of opisthosomal branchial appendages differs substantially from the ingression process in extant scorpions (Figures 2A, B and 3) [1,6,7], but tissue sections and TEM studies are needed to provide further clarification of cell activity in the formation of book gills. Book gill development has been studied only with light microscopy [6,7,87] and SEM [1] in the early stages of development; information is lacking about the ultrastructure of book gill and book lung formation in later instars before and after molts.

As pointed out by Fristrom [44], epithelial cells are transformed into nonpolarized mesenchyme cells during gastrulation, but most commonly the epithelial-derived cells have apical-basal polarity in their new location as did the parent cells. This means that caution should be used in inferring homology among similar patterns of epithelial cells. Wherever they occur in animals, epithelial cells tend to form sheets, layers and tubes, and their apical-basal polarity and organizational patterns are apparently very ancient [62]. Thus, organs with a similar epithelial structure are not necessarily indicative of a common ancestor (e.g., epithelial-derived exocrine glands in vertebrates and invertebrates, tracheae and bronchi, insect and spider tracheae).

The precursor cells for book gills are aligned in rows by hypodermal evagination [1,7] while ingression and alignment occur for scorpion book lungs (Figures 2A, B, 3, 5, 6, 7). Once positioned, the precursor cells have some similar activities in the development of book gill [1,7] and book lung lamellae: 1) cell proliferation, 2) shaping of cuticular structures as a result of cell growth and positioning relative to each other, 3) alignment of cells side-by-side into rows as commonly occurs in the hypodermis, 4) apical-basal polarity in these aligned cells like that in the hypodermis, 5) cell synthesis, transport and release of material for cuticle and 6) the cellular formation and cuticularization of space holders.

The results in this and earlier studies with SEM [1,27] thus provide evidence pro and con for book gill/book lung homology, depending on how much emphasis is given to the specific developmental differences and similarities. Page-like lamellae are the result for both respiratory organs, but both are formed from a cell type (epithelium) that typically forms ducts, tubes, layers and channels in a diversity of animals and organs.

### Epithelial morphogenesis

Numerous model systems (e.g., tracheae, zebrafish sense organs, bronchi, kidney tubules, vertebrate neural crest, blood vessels) are presently being used to study the cellular and molecular basis of epithelial morphogenesis [70-74]. Cell activity with some stages like those of book lung precursor cells occurs in an *in vitro *model using Madin-Darby canine kidney (MDCK) epithelial cells [73]. Starting from a cyst with a wall consisting of a single layer of polarized (apical surface inward) epithelial cells, cells can be stimulated to proliferate, ingress outward from the wall and align side-by-side. The apical surface of these aligned cells gradually produces a tubular lumen extending outward from the lumen of the original cyst. The resulting structure is somewhat like the air sacs extending anteriorly from the scorpion atrium.

Among the model systems, tracheal development in *Drosophila *is receiving much attention for the genetic determination of cell activity during tubulogenesis [74,88-90]. Some of the same genes and cell processes involved in the formation of fruit fly tracheae are implicated in the development of tubular structures in vertebrates [90]. Cellular and genetic research for fruitfly tracheae may be helpful for understanding book lung development in scorpions since there are similar processes of epithelial morphogenesis in the formation of these respiratory organs: 1) invagination of hypodermis in forming the atrium, 2) cell migration, 3) expression of apical-basal polarity, 4) alignment of cells side-by-side and presumably with formation of new cell-cell connections, 5) secretion from the apical surface to form a lumen and 6) emptying of the lumen to allow passage of air [74,76,91].

The development of arachnid book lungs is another example of epithelial morphogenesis with some features well suited for comparative studies. The book lungs are relatively large and tractable, even in embryos. There are 2, 4, or 8 book lungs in each individual [1,30,49,50]. Lamellar development is a continuing process in successive molts, and within each book lung the same cell processes are apparently repeated to make numerous lamellae [27]. Finally, lamellate respiratory organs are important for our understanding of evolutionary history and taxonomic relationships [11,30,49,50,75,92-94].

## Conclusions

The results herein show that some cellular activities are similar for book gill and book lung formation, but there are also important differences. The present investigation thus provides evidence for and against the hypothesis for book gill/book lung homology. The features of cell alignment and apical-basal polarity described herein are common among epithelial cells in animals, but the precursor cells for book gills and book lungs are distinctive in organizing themselves into a compact mass of many parallel, non-branching rows, i.e., the page-like pattern of alternating hemolymph and air or water channels.

The genetic and molecular basis of epithelial morphogeneis is presently receiving much research attention using model systems such as the tracheae of *Drosophila*. Book lung formation in scorpions is another example of epithelial cell morphogenesis, and many genetic and molecular features now being demonstrated in model systems in other animals may also be applicable for the development of book lungs.

## Competing interests

The author declares that he has no competing interests.
